# Genicular Artery Embolization: A Technical Review of Anatomy, Pathophysiology, Current Experiences, and Future Directions

**DOI:** 10.3390/jcm14062106

**Published:** 2025-03-19

**Authors:** Joseph M. Brown, Zachary T. Vandeveer, Danielle Cadoret, James J. Morrison, Younes Jahangiri

**Affiliations:** 1 Division of Interventional Radiology, Corewell Health West Michigan, Grand Rapids, MI 49503, USA; joseph.brown3@corewellhealth.org (J.M.B.); zachary.vandeveer@corewellhealth.org (Z.T.V.); danielle.cadoret@corewellhealth.org (D.C.); jmorrison@advancedrad.com (J.J.M.); 2 Department of Radiology, Michigan State University, East Lansing, MI 4882, USA

**Keywords:** genicular artery embolization, knee osteoarthritis, total knee arthroplasty

## Abstract

Genicular artery embolization (GAE) is an emerging minimally invasive procedure for managing knee osteoarthritis (OA), a condition affecting 365 million individuals globally. Initially developed to treat hemarthrosis, GAE selectively embolizes abnormal genicular vasculature, targeting synovial inflammation and reducing neoangiogenesis. This process alleviates pain and improves joint function, providing an alternative for patients with mild-to-moderate OA who are not candidates for surgical interventions due to comorbidities or other factors. Current evidence supports the use of GAE for patients with mild-to-moderate OA who experience persistent symptoms despite conservative treatments such as physical therapy, weight management, or intra-articular injections. The procedure effectively reduces pain, improves functionality, and provides sustained benefits. This review highlights the anatomical principles, procedural techniques, and patient selection criteria for GAE, as well as the clinical evidence supporting its safety and efficacy. It also explores potential future directions for research, including optimizing patient selection, evaluating long-term outcomes, and integrating GAE into routine OA management pathways.

## 1. Introduction

Degenerative joint disease is a common and disabling condition that most individuals will experience in their lifetime, with osteoarthritis (OA) being its most prevalent form. In 2019, an estimated 528 million people worldwide were living with OA, marking a 113% increase since 1990 [1]. This rise is partly attributed to the obesity epidemic, particularly in the United States, where obesity prevalence increased from 6.8% in 1980 to 22.4% in 2019, the highest rate globally [2]. The growing prevalence of obesity parallels a similar trend in OA worldwide. In the U.S. alone, OA imposes an annual economic burden exceeding USD 27 billion, with knee OA expected to contribute significantly as the population continues to age and obesity rates rise [3]. The condition arises from failed physiologic repair of cartilaginous damage, which can occur through a variety of seemingly mundane and routine events [4]. For example, such insults can be the result of repetitive occupational and recreational movements, including bending of the knee and heavy lifting with the lower extremities, as well as ligamentous and osseous injuries of the joint and congenital abnormal knee alignment [4]. Although common risk factors for knee OA include obesity, advanced age, and female sex, the susceptibility to an underlying insult that sparks the pathophysiologic cascade of OA reaches most individuals, which likely accounts for the dramatic global burden of disease and associated economic consequences [4].

Data indicate that 88% of individuals with OA are aged 45 or older, and more than half of those with knee OA will undergo total knee arthroplasty (TKA) in their lifetime [5,6]. As obesity and OA rates continue to rise, the number of TKA procedures is also expected to rise. However, many patients experience persistent pain and functional limitations if they are not suitable candidates for surgery due to comorbidities, preference to avoid surgery, or cannot afford TKA. Genicular artery embolization (GAE) is emerging as a potential alternative treatment modality. Recently, this technique has gained recognition as a safe and effective option for managing OA [7].

This narrative review aims to provide a comprehensive overview of the procedural technique, anatomical considerations, indications, patient selection criteria, and post-procedural assessment strategies for GAE.

## 2. History of Genicular Artery Embolization

GAE was first documented to treat spontaneous recurrent hemarthrosis following TKA [8,9]. Its utility in managing mild-to-moderate OA was first reported by Okuno et al. in 2015 [10]. In this initial pilot study, 11 patients were included, of whom 8 underwent GAE using temporary embolization with imipenem/cilastatin (IPM-CS) particles and 3 received embolization using 75 μm permanent embolic particles (Embozene; Varian Medical) [10].

Several small pilot studies conducted across Europe and North America subsequently yielded similar patient outcomes, sparking further investigation and interest [11,12,13,14]. A prospective study by Okuno et al. in 2017 demonstrated significant improvements in both pain and function post-procedurally, lasting up to two years from the initial treatment. These findings suggest a greater therapeutic longevity compared to intra-articular injections [11]. A few subsequent trials demonstrated similar results [12,13,14,15,16].

The GENESIS Trial, a prospective, single-center pilot study conducted in the United Kingdom (U.K.) released interim results in 2021 demonstrating the procedure’s safety, with only mild transient complications reported, as well as effective clinical responses at one year [17]. Shortly thereafter, results from the first randomized control trial, performed in the United States, were published by Bagla et al. in 2022 [18]. This study compared symptom reduction in participants with mild-to-moderate OA following GAE versus a sham control procedure, demonstrating clinically significant reductions in pain and disability after GAE [18]. Recently, the highly anticipated long-term follow-up data from the GENESIS Trial became available in 2024, reporting sustained therapeutic effects at 2 years without any additional long-term complications [19]. Furthermore, no additional operational complexities were observed in patients who proceeded to knee arthroplasty after GAE [19].

Currently, several registered clinical trials and prospective studies are assessing the role of GAE in OA, including those investigating novel embolic agents [20,21,22,23,24,25,26,27,28,29,30,31]. A summary of these studies is provided in Table 1. Notably, the phase 2 single-arm GAE-OA Trial, conducted in the U.S., recently published results demonstrating that GAE provides sustained pain relief at the two-year follow up in patients with moderate-to-severe knee OA, without significant adverse effects [32]. The GRAVITY Trial is an open-label, randomized control trial designed to compare the clinical outcomes of GAE versus conservative management for moderate-to-severe knee OA [26]. Additionally, it aims to identify changes in biomarkers and radiological findings in response to the procedure. The trial is expected to conclude in 2027 [26]. The GENESIS II Trial, a single-center, randomized control trial in the U.K., is comparing the clinical outcomes of GAE versus a sham control procedure, and the MOTION Trial, an international multicenter study, aims to evaluate clinical outcomes of GAE versus intra-articular corticosteroid injection in patients with mild-to-severe knee OA [23,24]. Both of these trials are in their recruitment phase.

## 3. Indications and Patient Selection

Since the introduction of GAE for the treatment of knee OA, the procedure has been recommended for patients with symptoms refractory to conservative approaches such as exercise, weight loss, physical therapy, and pharmacological management but who are either ineligible or unwilling to undergo surgical intervention [7,10]. Current guidelines recommend a gradual escalation in the intensity of conservative management strategies according to disease severity, disability, and pain, before progressing to minimally invasive procedures such as intra-articular injections for short term pain control [7,39].

Presently, there is limited research to suggest an association between baseline patient demographics or characteristics and the expected clinical response or duration of therapeutic effects of GAE. Early trials either did not include a subgroup analysis due to highly selected patient populations, lacked sufficient cohort sizes to perform meaningful subgroup analyses, or reported no statistically significant differences between clinical improvements and baseline patient characteristics [10,11,17,19,32,40,41]. However, promising clinical success rates for GAE have been documented in patients with OA disease burdens ranging from mild to severe, as defined either radiologically or clinically [12,13,14,40,42]. Clinically, studies have shown that patient-reported pain improves significantly more at 12-month follow-up in those with mild–moderate stages of the disease compared to those with severe OA [13,43]. Some studies suggest that patients with greater baseline pain severity and higher body mass index (BMI) tend to experience a greater reduction in symptoms post-procedurally and may be more responsive to the therapeutic benefits of GAE [44]. It has been suggested that patients exhibiting higher levels of pain catastrophizing—a psychosocial phenomenon in which an exaggerated negative appraisal of pain heightens pain perception and distress—at baseline perceived a greater reduction in pain after undergoing the GAE procedure [19].

The specific criteria for optimal candidates for GAE are still evolving. In general, inclusion criteria in clinical trials consist of patients >40 years of age with mild-to-severe knee osteoarthritis as defined by a Kellgren–Lawrence (KL) score of 1–4 on knee radiograph, persistent pain scored at ≥40 mm on the visual analog scale (VAS) despite conservative therapy for at least 3–6 months, and either ineligibility for or refusal of surgical intervention [10,11,13,17,19,32,40]. Despite small differences across studies, the overarching principle at this time suggests that patients with mild-to-severe disease and symptoms refractory to conservative management efforts for at least three months may derive clinical benefit from GAE, potentially delaying or avoiding surgical intervention. As with all clinical decisions, a multidisciplinary approach should be taken when considering candidacy for GAE, prioritizing treatments that best serve the patients autonomy and quality of life.

Suggested contraindications of GAE include severe peripheral arterial disease (PAD) due to risk compromising potentially collateral circulation of the lower extremity via the genicular arteries, active or suspected knee infection, renal dysfunction due to the risk of contrast-induced renal failure, a radiographically normal knee joint, and a history of fibromyalgia, autoimmune or inflammatory disorders as these patients may carry an alternative etiology of their knee pain [45]. A summary of these suggested contraindications, with the respective rationales, possible complications, and various strategies to minimize risk, are included in Table 2.

## 4. Assessment of Osteoarthritis Severity

Several scales have been consistently used across studies to create a standardized assessment of OA severity and response to treatment. These include the Kellgren–Lawrence score (KL), the Whole-Organ Magnetic Resonance Imaging Score (WORMS), the Western Ontario and McMaster universities Arthritis Index (WOMAC), and the Knee Injury and Osteoarthritis Outcome Score (KOOS). The KL and WORMS scales assess knee imaging findings of OA–KL specifically applies to knee radiographs, and WORMS to knee MRI. Plain radiography of the knee remains a cost-effective, quick, and key assessment of OA severity, and the original KL score was one of the first standardization attempts created for radiographic findings of arthritis in 1957 [46,47]. Since then, it has been used in several landmark osteoarthritis studies and development of radiographic OA atlases, has been repeatedly assessed to be a well-validated tool, and is currently the most widely used tool in assessing radiographic knee OA findings [48], which made it a practical choice for the vast majority of emerging GAE studies.

The incorporation of MRI evaluation is frequent in the current body of research investigating GAE for knee OA, as it measures changes in progression of features of joint destruction and allows for radiological assessment of whether interventions have been successful [49]. Whole-organ assessment became the popular option as it includes assessment of multiple features of the joint, which is key in accurately assessing OA severity [48]. There are a few options for whole organ assessment scales, including WORMS, the Knee Osteoarthritis Scoring System (KOSS), the Boston Leeds Osteoarthritis Knee Score (BLOKS), and the MRI Osteoarthritis Knee Score (MOAKS), which is a combination of WORMS and BLOKS. WORMS was the first and remains the most frequently applied semiquantitative scoring of knee OA in the literature; it was published in 2006 and has since been used extensively in epidemiological and longitudinal studies of knee OA [50]. It is the mainstay in GAE studies as it was the first available; its scoring system uses a regional rather than lesion-based approach, which gives a single score per region and allows for easier comparison with future studies; and it is the only scale that assesses subchondral bone attrition [50]. Although newer studies may move to using MOAKS in the future, in this review of GAE work up to date, WORMS has been the most consistently and widely used score and therefore has been the main MRI scoring system this paper will refer to.

Apart from objective imaging findings of knee OA features, WOMAC and KOOS allows standardized assessment of patient symptoms of knee OA, and interval patient-relevant outcome assessment of treatment effects. WOMAC is the original, recommended and most widely used osteoarthritis-specific scoring system in knee OA and GAE studies [51]. With its extensive use in clinical trials and longitudinal studies, it is one of the most reliable, valid and responsive tools in assessing knee OA and has allowed for direct comparison of clinical outcomes between early GAE trails [52]. However, it was developed for a more senior population with OA, and with GAE focusing on earlier intervention on younger, more active patients, the KOOS was needed to fill this gap. It is an extension of the WOMAC and includes categories assessing sport and recreation and more demanding activities in quality-of-life assessments [51]. Since its conception, it has been proven to be more sensitive and responsive in this younger and more active population and as such is used consistently with WOMAC in GAE studies [51].

## 5. Pre-Treatment Evaluation

Once a patient meets the inclusion criteria and is deemed a candidate for GAE, referral to the interventional radiology service can be placed along with the relevant clinical information, summarized in Table 3. A baseline assessment of both subjective and objective measures of disease burden should be obtained before the procedure.

The WOMAC Osteoarthritis Index and the VAS are useful validated tools to quantify pain levels before and after the procedure. The KOOS, developed from the WOMAC index, has been used in several studies to evaluate clinical response, as it specifically assesses changes in knee pain following intervention [7,11,17]. Assessing quality of life is another important aspect of the pre-treatment evaluation that can also be used for treatment effectiveness evaluation post-procedurally. OA-specific quality of life questionnaires such as the Osteoarthritis Knee and Hip Quality of Life questionnaire (OAKHQOL) [53] and OA quality of life (OA-QoL) [54] questionnaires have been developed and validated. A summary of these scores can be found in Table 4, and the KOOS and WOMAC assessment questionnaires are presented in Table A1 and Table A2, respectively. Clinically, the four quadrants of the knee should be palpated, and the level of pain documented to establish a baseline for post-procedural comparison. The four-quadrant knee pain assessment can even be performed before the embolization procedure and the painful areas can be marked with radiopaque beads (BB markers) on the skin for better targeting of the arterial territories contributing to the pain during angiography. A complete standard knee examination should be performed to assess the mobility and functionality of the knee joint and patella [55]. Additionally, patients’ use of pain medication should be recorded to monitor changes in usage after the procedure [19,55].

A recent weight-bearing knee radiograph is necessary to establish the baseline severity of the radiographic osteoarthritic changes, commonly assessed using the KL score based on joint space narrowing, sclerosis, and osteophyte formation graded from 0 to 4, with 0 being normal and 4 being severe OA [7,17,47]. Additionally, a recent knee MRI is required for several reasons [48]. First, it helps exclude other possible causes of pain that may require alternative treatment approaches. Second, it provides a baseline assessment of synovitis, synovial hypervascularity, and joint effusion using WORMS, which is used to monitor radiological treatment effectiveness post-procedure. WORMS is a comprehensive scoring system for evaluation of knee joint degeneration based on MRI features. In this scoring system, 14 different articular features, including cartilage, bone and bone marrow, osteophytes, menisci, ligaments, and synovitis, are assessed and scored in 15 different anatomical compartments of the knee. The total scores were summarized for four joint species (lateral and medial femorotibial joints, the patellofemoral joint, and the subspinous (S) region). Each region is assigned a total score, with a higher score showing more severe degeneration. The maximum total score for the entire knee joint is 332 [56]. Preoperative CT or MR angiography is not recommended, and anatomical mapping is typically performed during the initial diagnostic angiography at the start of the embolization procedure [7,17,48,55,57].

Relevant demographic data that should be documented include BMI, peripheral arterial disease (PAD) status, and risk factors for PAD. There is mixed evidence regarding the association between body mass index and treatment response, with some studies suggesting an increased risk of knee OA progression and a higher likelihood of treatment failure with higher BMI values [17,58]. This may necessitate alternative discussions or treatment approaches with the patient and orthopedic surgery service, as the effects of GAE may not be as long-lasting in this population. However, a discussion regarding the potential role of GAE in managing these patients should still be initiated [11]. The presence and severity of PAD are important considerations, as the genicular arteries often serve as the dominant collateral vessels in advanced PAD. This may impact the extent of vessel pruning that can be safely targeted during the procedure [17,59].

When obtaining consent for GAE, the discussion should include the reason for treatment, procedural details, risks, and benefits, as is standard for any minimally invasive procedure. Specifically, it is important to address the patient’s expectations regarding pain improvement. Symptomatic relief may be perceived within hours to weeks following the procedure. The specific risks of GAE should also be discussed. A more detailed discussion of procedural complications is provided in a subsequent section in this paper. Generally, major complications have been extremely rare, and mild-to-moderate complications include access site bleeding, hematoma, and pseudoaneurysm, as well as the potential for non-target embolization, particularly affecting the overlying cutaneous tissue. This complication can range from mild erythema to skin necrosis, with cutaneous erythema or discoloration being the most commonly documented complication in the current literature, as described later in this manuscript [7,13,14]. Patients should also understand that undergoing GAE does not eliminate the possibility of requiring future surgical intervention, and risk factors such as PAD, obesity, and sedentary lifestyle may increase this likelihood.

**Table 4:** Clinical knee osteoarthritis scoring systems [47,51,53,54,56,60,61,62,63].

Name	Type	Use	Scoring
WOMAC ^1^	Questionnaire	Knee OA-specific Measures pain, stiffness, physical function Most commonly used instrument in knee OA	Total score: 0–20 for Pain, 0–8 for Stiffness, 0–68 for Physical Function The higher the score, the worse the OA Three domains, each scored separately, 24 questions total Scores not aggregated, encouraged to interpret separately
KOOS ^2^	Questionnaire	Knee OA-specific Similar measurements to WOMAC but has additional domains relating to sport and recreation, knee-related quality of life Developed for younger/more active patients with knee injury More sensitive to change over time than WOMAC	Total score: 0–100 for each domain 0 = extreme problems, 100 = no problems Five domains each scored separately, 42 questions in total Sum of the Likert scale scores for each domain, then converted to 0–100 scale. Scores not aggregated, encouraged to interpret separately
VAS ^3^	Continuous horizontal/ vertical 10 cm visual scale	For measuring pain intensity at a specific moment in time Patient picks the intensity that most closely matches their level of pain	Scale commonly between 0 (no pain) and 10 (worst pain imaginable) with anchors (numbers/descriptors/facial expressions) spread evenly Numbers/descriptors at intermediate points not encouraged One question test
K-L ^4^	Radiographic Classification Scheme	Specifically for standardized grading of knee OA on plain films	Grades 0–4, with 0 being no OA and 4 being severe radiographic OA based on joint space narrowing, sclerosis, and osteophyte formation
WORMS ^5^	MRI Classification Scheme	Specifically for grading osteoarthritis on knee MRIs Semi-quantitative, multi-feature assessment “Whole organ”, meaning it includes evaluation of articular tissue and its role in OA—cartilage, menisci, and ligaments, not just ossified structures	Scores assess 14 different articular features, e.g., cartilage signal, osteophytes meniscus integrity, synovitis, etc. Each feature has its own scale, increments, score totals to reflect specific clinically relevant changes; e.g., osteophytes are assessed on an 8-point scale, ACL are scored as 0 (intact) or 1 (torn), menisci are divided into parts and each part scored from 0 to 4, etc.
OAKHQOL ^6^	Questionnaire	Evaluating knee and hip osteoarthritis-specific quality of life	43-item questionnaire assessing 5 dimensions of quality of life, including physical activities, mental health, pain, social support, and social functioning.
OA-QoL ^7^	Questionnaire	Evaluating quality of life in patients with osteoarthritis	22-item unidimensional true/false questionnaire for patient-based quality of life report with good demonstrated psychometric properties

^1^ Western Ontario and McMaster universities Arthritis Index (Appendix A Table A2). ^2^ Knee injury and Osteoarthritis Outcome Score (Appendix A Table A1). ^3^ Visual analog scale. ^4^ Kellgren–Lawrence Score. ^5^ Whole-Organ Magnetic Resonance Imaging Score. ^6^ Osteoarthritis Knee and Hip Quality of Life 2.2. ^7^ Osteoarthritis Quality of Life.

## 6. Pathophysiology

To appreciate the therapeutic principles of GAE, it is essential to understand the biochemical mechanisms and inflammatory cascade of OA [7]. Traditionally, the prevailing hypothesis has been that chronic mechanical “wear and tear” is the primary underlying pathophysiology of OA. However, advancements in technology have provided more recent evidence at the epigenetic, molecular, and cellular levels, suggesting that chronic, low-grade inflammation plays a significant role in disease progression and symptomatology [42,63]. This refined understanding of OA pathophysiology has contributed to the development of potential treatment options and preventive measures.

**Figure 1:**
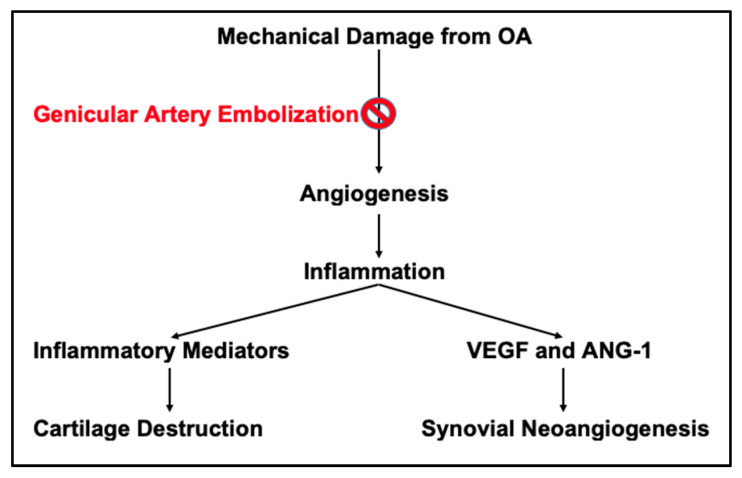
Pathogenic cascade of OA with inflammatory mediators and synovial neoangiogenesis. GAE targets the areas of hyperemia, impeding the arrival of destructive inflammatory and neurovascular mediators, thereby inhibiting further cartilage destruction and synovial neoangiogenesis [7].

At a macroscopic level, stress on the articular surfaces from repetitive activity or bearing excessive body weight leads to chronic inflammation and the degradation of protective joint cartilage. This process inevitably triggers a cascade of inflammatory mediator production, compensatory synovial hypertrophy, angiogenesis, and further osteoarticular destruction [64]. A simplified schematic demonstration of the proposed pathophysiology is presented in Figure 1 [7]. This inflammatory cascade and biochemical mechanism result in synovial neoangiogenesis, accompanied by the release of vascular endothelial growth factor (VEGF) and Angiopoietin-1 (Ang-1) [64]. These pro-angiogenic factors drive neovascularization and innervation of an otherwise aneural articular surface [64,65]. As inflammatory mediators and biochemical cytokines infiltrate target tissues, they initiate and exacerbate hyperplasia, inflammation, and cartilage destruction, promoting the progression of disease burden [64,65].

At the cellular level, macroscopic inflammatory changes trigger alterations in epigenetic regulation through deoxyribonucleic acid (DNA) methylation, histone modifications, and micro-ribonucleic acid (miRNA) activity. These modifications promote hypertrophic, proinflammatory, and even self-destructive cellular mediators and behaviors [63]. This vicious cycle of ongoing inflammation and cellular destruction is believed to be responsible for the progressive pain associated with OA. The perception of pain arises through mechanical and biochemical stimulation of nociceptors within the synovium, subchondral bone, menisci, ligaments, tendons, and periarticular fat pads [63]. Multiple proinflammatory chemokines and immune mediators interact with these nociceptors at biochemical level to mediate pain transmission through peripheral nerve fibers via the nerve growth factor (NGF), calcitonin gene-related peptide (CGRP), chemokine C-C motif ligand 2 and receptor 2 (CL2/CCR2), and tumor necrosis factor (TNF)-α signaling pathways [63]. Additionally, the chronic inflammatory environment surrounding the osteoarthritic joint has been shown to sensitize nerves and neural pathways, both peripherally and centrally [63,66,67]. This sensitization can lead to an enhanced perception of noxious stimuli and the perception of pain in response to an otherwise non-painful stimuli, known as hyperalgesia and allodynia, respectively, resulting in debilitating symptoms for affected patients [63,66,67].

GAE aims to disrupt this cascade through selective embolization of abnormal microcirculatory genicular arterial branches. By reducing the vascular supply that delivers proinflammatory mediators, GAE interrupts the destructive cascade at the molecular and cellular levels [42].

## 7. Anatomical Considerations

### 7.1. Anatomy of the Genicular Anastomosis

**Figure 2:**
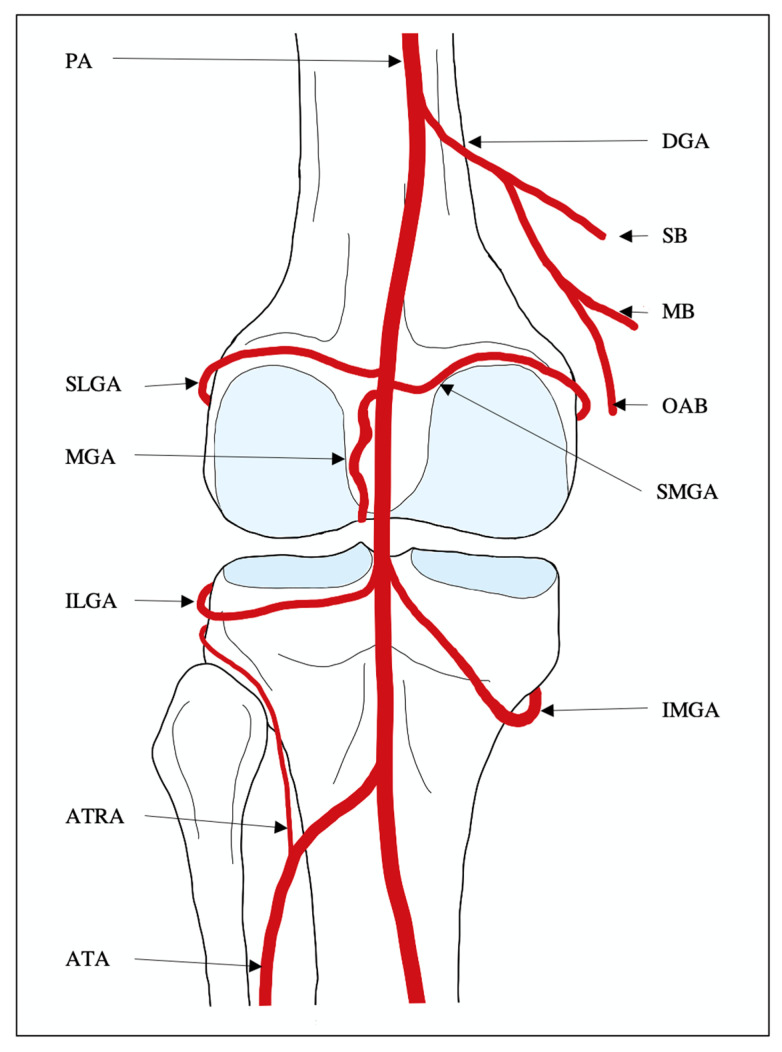
Schematic demonstration of the classic configuration of the genicular anastomosis. From the posterior view of the left knee, the collateral vascular supply of the genicular anastomosis including the popliteal artery (PA), descending genicular artery (DGA), saphenous branch of the descending genicular artery (SB), muscular branch of the descending genicular artery (MB), osteoarticular branch of the descending genicular artery (OAB), superior medial genicular artery (SMGA), superior lateral genicular artery (SLGA), middle genicular artery (MGA), inferior medial genicular artery (IMGA), inferior lateral genicular artery (ILGA), anterior tibial recurrent artery (ATRA), and anterior tibial artery (ATA) [64]. Reprinted from Genicular Artery Embolization: A Review of Essential Anatomic Considerations, 35:4, Shu Liu, David Swilling, Elizabeth M. Morris, William Macaulay, Jafar Golzarian, Ryan Hickey, Bedros Taslakian, Pages 487–496, 1 Apr 2024, with permission from Elsevier [69].

The knee joint receives its vascular supply from the genicular arteries, a complex and often variable arcade of vessels originating from the distal femoral, popliteal and anterior tibial arteries. These genicular arteries form a collateral network commonly known as the genicular anastomosis [68]. In the classic anatomical configuration of the genicular anastomosis, five primary vessels consistently arise from the popliteal artery, providing the majority of the arterial supply. These include the superior lateral genicular artery (SLGA), superior medial genicular artery (SMGA), middle genicular artery (MGA), inferior lateral genicular artery (ILGA), and inferior medial genicular artery (IMGA) [68]. Additionally, arterial contributions from branches of the distal superficial femoral, popliteal, and anterior tibial arteries are frequently encountered [68]. A thorough understanding and intraoperative appreciation of the vascular anatomy and potential anatomical variants is essential for procedural efficiency and to minimize complications [7,69]. The classical configuration of the genicular anastomosis is illustrated in Figure 2.

The SLGA originates from the anterolateral aspect of the popliteal artery, cranial to the meniscus, and travels laterally between the lateral femoral epicondyle and the iliotibial tract [69]. Along its course, it gives off several muscular and osseous branches before extending anteriorly around the femur, where it bifurcates into the patellar and deep articular branches, which are major arterial suppliers of the lateral femoral condyle [69,70]. In addition to vascularizing the lateral compartments of the patellofemoral and the knee joints, the SLGA supplies the skin of the lateral knee, the lateral collateral ligament, the lateral head of the gastrocnemius muscle, and the distal regions of the vastus lateralis and iliotibial tract [69,70].

The SMGA originates from the anteromedial aspect of the popliteal artery, cranial to the level of the meniscus. It initially arches superomedially between the medial femoral epicondyle and the tendons of the adductor magnus and vastus medialis muscles before coursing anteriorly and bifurcating into superior and inferior branches [69]. Ultimately, the SMGA supplies the medial compartments of the patellofemoral and the knee joints, as well as the medial collateral ligament and the distal regions of the vastus medialis, semimembranosus and semitendinosus muscles [71]. Although the SMGA is thought to contribute to cutaneous supply, studies suggest it has minimal clinical significance in many individuals [72].

The MGA originates from the anterior surface of the popliteal artery, often immediately caudal to the origin of the SLGA [69,73]. It penetrates the posterior knee joint capsule between the femoral condyles and serves as the dominant supplier of internal joint structures, including the cruciate ligaments, synovium, menisci and femoral and tibial epiphyses [73].

The ILGA typically originates from the anterolateral aspect of the popliteal artery, near the level of the meniscus. It courses laterally between the lateral tibial plateau and the posterolateral ligamentous complex, supplying the fibular head and surrounding soft tissues [69,72]. As it travels anteriorly around the tibia, the ILGA supplies the lateral compartments of the patellofemoral and the knee joints, as well as the skin of the lateral knee, the infrapatellar fat pad, and the anterior cruciate ligament [69,74].

The IMGA arises from the anteromedial aspect of the popliteal artery near the level of the meniscus and courses inferomedially adjacent to the popliteus muscle [69,75]. It then travels around the medial tibial condyle and beneath the medial head of the gastrocnemius muscle before passing anteriorly beneath the medial collateral ligament (MCL), ascending towards the tibial tuberosity [69]. The IMGA supplies the medial compartment of the patellofemoral and the knee joints, as well as the skin of the medial knee, the infrapatellar fat pad, the popliteus muscle and the anterior cruciate ligament [75].

In addition to the five primary genicular arteries, tributaries of the distal superficial femoral (SFA) and anterior tibial arteries (ATA) can contribute to the genicular anastomosis and should be considered when performing GAE [68,69]. The descending genicular artery (DGA) originates from the medial surface of the distal superficial femoral artery, adjacent to the adductor hiatus [69]. It descends the medial thigh and classically divides into three terminal branches: the saphenous branch, the muscular branch, and the osteoarticular branch [69,71,76]. The saphenous branch travels superficially alongside the saphenous nerve, supplying the skin of the distal medial thigh and the superomedial knee, as well as the saphenous nerve, sartorius muscle, and regions of the proximal tibia [69,72]. The muscular branch originates perpendicularly from the common DGA origin and supplies in the muscles in the distal medial compartment of the thigh, including the rectus femoris, vastus medialis, vastus intermedius, adductor magnus, sartorius, and gracilis muscles [69,71,72]. The osteoarticular branch descends through the deep distal medial thigh, supplying the distal vastus medialis muscle before coursing anteriorly toward the adductor tubercle. It serves as a dominant vascular supply to the medial femoral condyle, making it a common embolization target in GAE for medial compartment OA [74]. Finally, the anterior tibial recurrent artery (ATRA) originates at an acute angle from the proximal segment of the anterior tibial artery (ATA), just caudal to the knee joint [69,77]. It ascends through the lower leg within the tibialis anterior muscle, providing collateral supply to the lateral knee joint, the proximal tibia and fibula, and the patellar tendon [77].

### 7.2. Anatomical Variations

**Figure 3:**
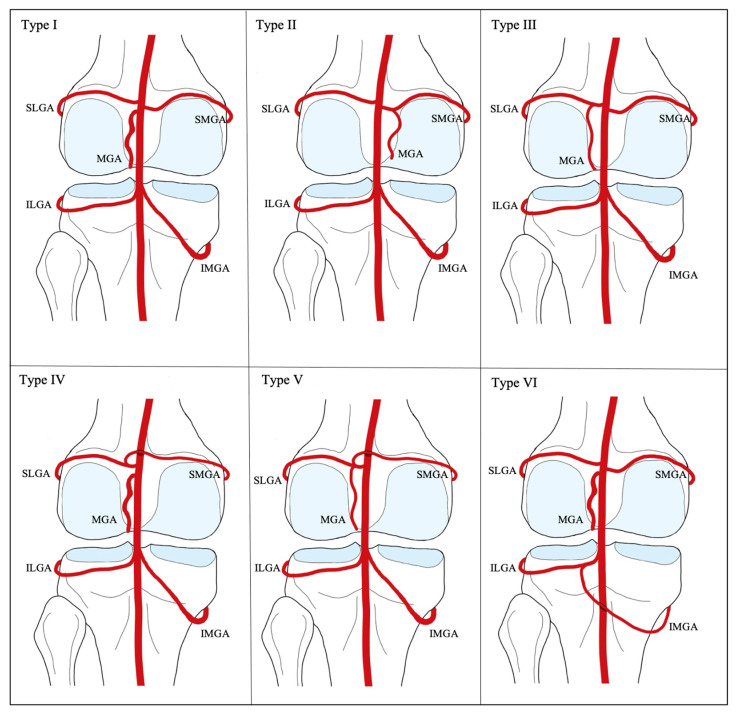
Sighary et al.’s classifications of anatomical variations of genicular anastomosis. Type I: The classically described configuration where all five genicular arteries have an individual origin from the popliteal artery (Callese Type 2). Type II: Common origin of the MGA and SMGA (Callese Type 4). Type III: Common origin of the MGA and SMGA (Callese Type 1). Type IV: Common origin of the SLGA and MLGA. Type V: Common origin of the SLGA, SMGA, and MGA (Callese Type 3). Type VI: Common origin of the IMGA and ILGA [82,83].

**Figure 4:**
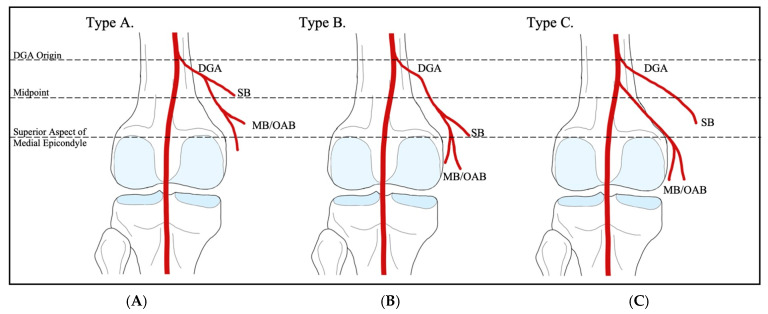
Sighary et al.’s classifications of anatomical variations of the descending genicular artery. (**A**) Type A—Initial bifurcation of the saphenous branch (SB) and the muscular/osteoarticular branch (MB/OAB) occurs above the midpoint between the DGA origin and the superior aspect of the medial femoral epicondyle. (**B**) Type B—Initial bifurcation of the SB and MB/OAB occurs below the level of the midpoint. (**C**) Type C—Separate origins of the SB and MB/OAB [83].

The branching pattern of the genicular anastomosis exhibits highly variable anatomical configurations, which are crucial to recognize intraoperatively to avoid non-target embolization and enhance procedural efficiency [7,69]. The medial compartment of the knee has been shown to bear a higher burden of disease; therefore, the medial vascular supply may be more frequently targeted during GAE, warranting close attention to anatomical variants of the contributing arteries [7,78,79,80,81]. Several cadaveric and radiological studies have investigated the anatomical branching patterns of the genicular anastomosis, proposing different classification systems [71,78,82,83,84,85]. Among the most robust studies are a 2022 cadaveric study by Sighary et al. and a 2023 study by Callese et al., which utilized intraoperative GAE cone-beam computed tomography scans. These classification systems are outlined in Table 5 and illustrated in Figure 3 and Figure 4 [69,82,83].

In the study by Sighary et al., the popliteal arteries of 204 cadavers were dissected, with 196 (96%) fitting into six specific classifications of genicular artery branching patterns [83]. This classification scheme designates Type 1 as the configuration with independent branches of the SLGA, SMGA, MGA, ILGA, and IMGA—consistent with the traditionally described anatomical pattern—and was found in 28% of specimens [83]. The remaining categories describe variable common origins of the superior and middle genicular arteries, with independent popliteal origins of the two inferior genicular arteries [83]. Additionally, this study proposed a classification system for the DGA, based on the craniocaudal location of its initial bifurcation, where the saphenous branch divides from the musculocutaneous and osteoarticular branches (Figure 4) [83]. The most common configuration, Type B, features a bifurcation distal to the midpoint between the DGA origin and the superior aspect of the medial epicondyle of the femur, accounting for 72% of the specimens [83].

The study by Callese et al. provided a retrospective intraoperative cone-beam computed tomography analysis of 205 patients undergoing GAE [82]. Four categories of the DGA anatomy were proposed, with 77% of cases exhibiting the standard bifurcation into the saphenous and musculocutaneous/osteoarticular branches [82]. Regarding the superior genicular arteries, four branching patterns were identified, the most common being a shared origin of the SLGA and MGA, observed in 56% of the cases [82]. The classic configuration, in which all superior genicular arteries have independent origins, was encountered in 22% of cases in this study [82]. In addition, two branching patterns of the inferior genicular arteries were described, though 99.5% exhibited independent origins. Notably, the study also identified a superior patellar artery in 85% of the cases, which contributed vascular supply to the joint space in all instances where it was observed [82].

## 8. Procedural Technique

GAE is typically an elective, outpatient procedure. All preprocedural steps for routine angiography should be followed, including basic laboratory studies, preprocedural fasting according to conscious sedation guidelines, appropriate management of oral anticoagulation medications according to arterial puncture criteria, and obtaining informed consent before the procedure [86]. Anticoagulation management is particularly pertinent, as most of these medications will need to be temporarily discontinued at variable intervals before the procedure.

Vitamin K antagonists, such as warfarin, are commonly used for oral anticoagulation, but several newer direct oral anticoagulants (DOACs) are now frequently prescribed. These include direct factor Xa inhibitors—betrixaban, apixaban, rivaroxaban, and edoxaban—as well as direct thrombin inhibitors, such as dabigatran and argatroban [87,88]. While these newer targeted anticoagulants offer advantages, their use and discontinuation before procedures require careful consideration. In emergent cases, reversal agents may be necessary [89]. For low-risk procedures in patients considered to have a low risk of bleeding, anticoagulation does not need to be interrupted [89]. However, for moderate- to high-risk procedures, withholding practices and anticoagulation interruption timing vary by medication [89]. Edoxaban and rivaroxaban should be withheld for at least 24 h before a moderate- or high-risk procedure, whereas apixaban and dabigatran should be held 24 h before moderate-risk interventions and 48 h before high-risk bleeding procedures [90,91]. The Society of Interventional Radiology has provided additional guidance on anticoagulant management timelines, as summarized in Table 6 [92].

Before entering the procedure room, the performing physician should conduct a physical examination of the affected knee. The area of pain and pathological concern can be marked with a metallic marker for fluoroscopic reference for targeting the contributing vascular tributaries [42]. The IR suite should be properly prepared for lower-extremity angiography, ensuring that all anticipated resources are readily available [86]. It is common for patients to receive 2 g cefazolin IV, 30 mg ketorolac IV, and 10 mg dexamethasone IV before or during the procedure [86]. Little et al. describe the use of an ice pack on the skin surface in the pre-procedural setting to induce temporary vasoconstriction at the anticipated embolization site, reducing the risk of non-target cutaneous branch embolization [17]. Notably, in their study, the incidence of non-target cutaneous branch embolization was 11% when using an ice pack, compared to 65% reported in the study by Bagla et al. and 57% in the study by Okuno et al. [11,12,17].

The GAE procedure is commonly performed under minimal or moderate sedation with local anesthesia as per institutional guidelines and patient preferences. Patients should be advised to fast for at least eight hours prior to any procedure requiring sedation [98]. The American Society of Anesthesiologists (ASA) describes sedation in the following categories:

1. Minimal sedation (“anxiolysis”): A drug-induced state in which patients respond normally to verbal commands [99]. While physical coordination and cognitive function may be impaired, airway reflexes, ventilatory function, and cardiovascular function remain unchanged [99]. Anxiolysis is typically achieved with a single oral dose of a sedative or analgesic before the procedure [99].
2. Moderate sedation/analgesia (“conscious sedation”): It is a drug-induced reduction in consciousness during which patients respond purposefully to verbal commands, either alone or with light tactile stimulation [99]. The patient maintains their airway and spontaneous ventilation without assistance [99]. Cardiovascular function remains stable, as IV sedatives and/or analgesics are carefully titrated throughout the procedure [99].
3. Deep sedation: A drug-induced state of reduced consciousness where patients cannot be easily aroused but still respond purposefully to repeated or painful stimulation [99]. Ventilatory function may be impaired, and airway support such as intubation or mechanical ventilation may be required. However, cardiovascular function is typically maintained through IV titration, similar to moderate sedation [99].
4. General anesthesia: A drug-induced loss of consciousness in which patients do not respond to painful stimulation and cannot maintain their airway independently. As a result, intubation with positive-pressure ventilation is necessary to ensure adequate respiration.

For vascular access, ipsilateral antegrade femoral artery access is recommended as the first-choice puncture site when feasible, depending on body habitus [86]. Alternatively, retrograde contralateral femoral artery access or ipsilateral radial artery access may be considered based on the operator and patient preference, institutional setup and availability of radial-specific access instruments [42]. The most technically challenging option is ipsilateral retrograde pedal access, typically performed via the posterior tibial or dorsalis pedis artery. This approach is generally reserved for cases in which femoral or radial access is not feasible [100].

**Figure 5:**
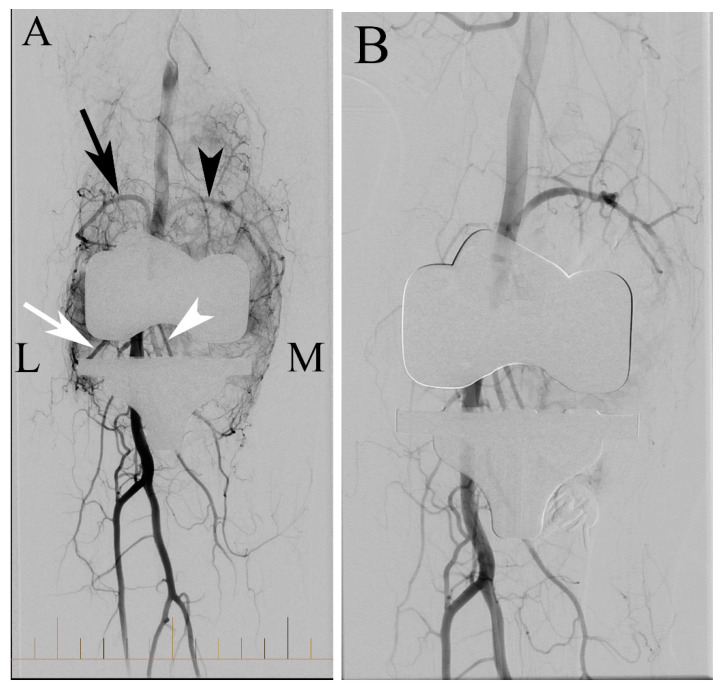
Right knee angiography in a 57-year-old female with hemarthrosis and recurrent painful knee approximately 3 years after total knee arthroplasty. Note significant periarticular hyperemia in the territories of lateral superior (black arrow), lateral inferior (white arrow), medial superior (black arrowhead), and medial inferior (white arrowhead) genicular arteries in the pre-embolization digital subtraction angiography (**A**). After selective embolization of the respective branches with 100–300 μm and 300–500 μm embolic beads to near stasis, there is a significant reduction in periarticular hyperemia on post-embolization digital subtraction angiography (**B**). On post-embolization follow-up, there was a significant reduction in the patient’s knee pain, and there was no recurrence of hemarthrosis.

Once an intravascular catheter is placed, angiography is performed from the distal superficial femoral artery, capturing early and delayed digital subtraction angiography (DSA) images to identify and target the index genicular artery for embolization. The abnormal target arteries exhibit hypertrophy and a “tumor-blush”, as shown in Figure 5 [42].

Previously placed radiopaque pain localizers can help guide the interventionist toward the abnormal genicular circulation and potential embolization targets [7]. Intra-procedural cone-beam computerized tomography (CBCT) of the knee, combined with simultaneous contrast injection via a power injector, can assist in mapping the genicular arteries, identifying variant anatomy, and recognizing branches at risk for non-target embolization [86]. Selective DSA in the target genicular artery is used to identify and avoid branches that may lead to non-target embolization of cutaneous or collateral branches [86]. Embolization is typically performed via a 1.7–2.4 Fr microcatheter using small-sized particles. Imipenem/cilastatin (IPM-CS) mixed with contrast (0.5 g of IPM-CS in 5–10 mL of iodinated contrast agent) was the first embolic agent used for this purpose due to its natural crystalline structure and temporary embolization characteristics. Alternatively, Embozene microspheres (Varian, Palo Alto, CA, USA; 75 μm in 2 mL of contrast agent) were used in patients with allergies to IPM-CS [10]. Studies have shown that IPM-CS has lower necrotic effects, even with complete occlusive embolization, compared to the nearly 100% necrotic effects observed with permanent embolics such as Embozene [101]. However, IPM-CS is not approved for GAE by the United States Food and Drug Administration (FDA) [42]. IPM-CS has a particle size of 10–70 μm and is the most commonly used embolic agent for GAE in OA treatment outside the U.S. [102]. In recent years, 75–100 μm permanent embolic particles have demonstrated successful outcomes for GAE in the treatment of OA, with no significant difference in clinical results when compared to IPM-CS [11,13]. These include the following:

- Embozene microspheres (75–100 μm) (Varian Medical Systems, Palo Alto, CA, USA);
- Embospheres (Merit Medical Systems, South Jordan, Utah; 100–300 μm);
- HydroPearl (Terumo, Tokyo, Japan; 200 μm);
- Polyvinyl alcohol (PVA, 10–70 μm) [7].

Furthermore, patients who underwent embolization with permanent particles reported a greater mean decrease in VAS and WOMAC scores at one month compared to those embolized with IPM-CS [103].

Understanding the embolization endpoint for GAE is crucial, as pruning hypervascularity while maintaining arterial flow differs from the common embolization goal of achieving complete stasis [86]. Technical success is not defined by complete stasis but rather by the resolution of distal hypervascularity while maintaining a patent parent genicular artery and branches on DSA, indicating an adequate embolized volume for GAE [86]. A slow injection of embolic beads under continuous fluoroscopic visualization is crucial to ensure proper endpoint detection [69]. Intermittent test contrast injections with DSA between each 0.2–0.4 mL injection of embolic material are advised to evaluate the target vessel’s robustness, monitor embolization speed, and prevent reflux, reducing the risk of non-target embolization [69,86]. Selective power injections immediately after embolization may theoretically displace particles through anastomoses and should be avoided [69].

Once embolization is complete and the catheters are removed, hemostasis can be achieved with direct manual pressure at the access site for at least 15 min, with gradual release of pressure over another several minutes. Alternatively, a vascular closure device can provide immediate and more secure closure of the arteriotomy site [7]. On average, the GAE procedure may take approximately 1–2 h in an outpatient setting, though duration may vary depending on procedural difficulty and operator experience [7]. Complete bed rest in a supine position for 4–6 h, along with instructions to avoid straining for 24–48 h, is recommended, particularly when hemostasis has been achieved through manual compression. Most patients can be discharged within 4 h after the procedure [15]. Discharge instructions vary by institution, but most advise patients to avoid heavy lifting, straining and strenuous exercise for at least 24–48 h. Laxative agents can be recommended prior to the procedure to decrease the risk of straining, especially in those with constipation. For patients with bilateral OA, treatment of the contralateral knee can be performed if indicated [86]. While specific guidelines regarding the interval between embolization of different knees have not been rigorously studied, theoretically, the contralateral knee can undergo GAE once the initial knee has had sufficient time for pain reduction and access site healing, and any potential complications have resolved.

## 9. Post-Treatment Follow-Up

There is a wide range of follow-up practices and protocols, but most commonly, patients are seen within one week of the procedure to assess for acute complications. This includes examining the skin overlying the treated knee for discoloration, ulceration, or necrosis, as non-target cutaneous embolization is the most frequently documented adverse effect in the literature [7,17,57]. Although osteonecrosis is a theoretical adverse effect of GAE, it is extremely rare, and permanent necrosis has not been reported in the literature. Possibility of osteonecrosis following GAE has been documented in only one study, where 3 out of 17 patients developed non-specific osteonecrosis-like areas on MRI one month post-GAE; however, all cases resolved within six months [104]. The only other reported case involved one patient (out of thirty-one) who developed aseptic necrosis of the femoral condyles following GAE performed for recurrent hemarthrosis after TKA [9]. While rare, the potential severity of osteonecrosis warrants high clinical suspicion. Post-procedural symptoms of osteonecrosis include localized pain disproportionate to the expected recovery and possible flexion contracture due to secondary muscle spasm [105,106]. Additionally, the access site should be assessed for infection or hematoma, and a brief neurological exam should be performed to rule out paresthesias or progression of neurological deficits [11,12,13,14,17].

Long-term follow-up includes periodic reassessment of the patient’s pain and functionality scores (WOMAC, VAS, and/or KOOS) or quality-of-life measures (OAQoL or OAKHQOL questionnaires) to evaluate the clinical response to GAE [11,12,13,14,17]. Common follow-up intervals include 1 month, 3–4 months, 6 months, and then every 6–12 months for up to 2–4 years [11,12,44]. Adjunct therapy usage, particularly pain medication, should be monitored, as GAE has been shown to reduce medication dependence, allowing for appropriate adjustments to controlled prescriptions [11,12,13,14,17]. Knee radiographs may be obtained to monitor radiographic disease progression if clinically indicated. In cases of treatment failure, repeat GAE may be considered after 12 months from the initial procedure [7,107].

Currently, the literature defines clinical success as at least a 50% reduction in pain, as measured by WOMAC scores, or at least a 10-point increase in the Knee injury and Osteoarthritis Outcome Score (KOOS). However, the time endpoint for clinical success varies between studies. For example, Correa et al. uses these criteria to define success at three months post-procedure, and sustained success at twelve months post-procedure [55]. Alternatively, Padia et al. defined clinical success as meeting those same criteria at 12 months post-procedure [15].

## 10. Complications

The complications experienced by patients after undergoing GAE are typically minor, fleeting, and frequently related to the selected embolic material [7]. The most commonly reported complication is transient cutaneous erythema, which occurs in approximately 12% of cases [12]. The incidence and duration of erythema are higher with permanent embolics (63%, lasting 1–3 months) compared with IPM-CS (2.5%, lasting 3 weeks) [11,12,13,14,17]. Further data suggest a higher incidence of overall complications with permanent embolics (Embozene, PVA, and Embospheres) than with temporary embolics (IPM-CS). Skin discoloration has been observed when using particle sizes smaller than 100 μm, and other studies report a high incidence of transient cutaneous erythema with microspheres <300 µm, as smaller embolics travel to more distal arterial branches [9,12,57]. In response, some authors now recommend avoiding embolic particles < 300 µm to minimize transient cutaneous complications [9,57].

Less commonly reported complications include access site hematomas (10%), plantar sensory paresthesia (1.1%), and mild temporary fevers (0.55%), all of which were transient, resolving in up to 2 weeks period [11,12,13,14,17]. Plantar paresthesias have been observed with smaller embolic particles (~75 μm diameter) and are believed to result from non-target embolization of the medial plantar nerve [12]. Some proceduralists advocate for the use of intravenous NSAIDs and corticosteroids to alleviate post-embolization syndrome (PES), a constellation of symptoms including pain, fever, nausea, and vomiting [15] seen up to 72 h after procedure. Although osteonecrosis is considered a serious theoretical complication of GAE, in practice, it has been an extremely rare entity, and there is no evidence of permanent necrosis in studies with MRI scans performed two years after the procedure [9,11].

Concerns have been raised regarding GAE’s potential tissue hypoperfusion and its adverse impact on wound healing if knee surgery is required. However, GAE specifically targets minuscule microvessels and aims to prune neoangiogenic vessels that contribute to OA progression, rather than affecting normal circulation. As a result, GAE has not been shown to increase surgical complexity or impair healing following arthroplasty in the long-term results of the GENESIS trial, the only clinical trial that has studied the topic [19].

## 11. Comparative Effectiveness

As previously discussed, GAE for the symptomatic treatment of knee OA is a relatively novel therapy that has gained attention in the international literature over the past decade, demonstrating promising technical and clinical results [7,10,11,12,13,14,17,19,108]. Currently, guidelines recommend a multifaceted approach to managing knee OA, beginning with non-pharmacological strategies, such as exercise, weight loss, transcutaneous electrical nerve stimulation, and orthopedic aids, alongside pharmacological treatments, including oral or topical analgesics and intra-articular injections [109]. Surgical referral is typically reserved for patients with symptomatic knee OA who experience a significant impact on quality of life and are refractory to the aforementioned conservative management options [109]. Despite increasing recognition in the clinical community, direct comparisons between GAE and alternative therapies remain limited in the current literature, with relatively variable results. More comprehensive research regarding the efficacy, duration of symptom relief, and risk of complications is needed to generate accurate and reliable comparisons to alternative treatment options. A comprehensive list of completed and active prospective studies and clinical trials is presented in Table 1 and reviewed from a historical perspective. In this section, a more detailed review of the results of these studies will be provided.

### 11.1. Conservative Management

Few studies have directly compared the clinical effectiveness of GAE to sham control procedures in which participants were allowed to continue concurrent conservative therapies post-procedure [18,40,81]. Two of these studies demonstrated either clinically or statistically significant pain reduction in the embolization group during short-term follow-up (between 4 and 12 months post-procedure) [18,81]. A third study conducted a subgroup analysis, comparing sham control procedures to participants who underwent single-vessel, incomplete, and complete embolization [40]. This analysis yielded progressive improvements in pain, functionality, and quality of life, with increasing benefits from single-vessel embolization to complete embolization, suggesting a possible dose–response effect [40].

A recent meta-analysis reported a 27% and 65% reduction in the number of patients using opioids and non-steroidal anti-inflammatory drugs (NSAIDs), respectively, after undergoing GAE [110].

Several ongoing clinical trials aim to provide further comparative data on GAE’s effectiveness versus sham procedures. The GENESIS II and LIPIOJOINT2 trials are currently recruiting participants to evaluate the clinical benefits of GAE in OA and its impact on reducing analgesic use post-procedure [22,23]. Each of the research groups involved in these trials have previously published the results of their initial trials. In the GENESIS Trial, statistically significant improvements in all KOOS subcategories, except for function in daily living, were demonstrated at 6-week through 1-year follow up, with nearly half of the participants experiencing improved symptoms sustained through 24 months [17,19]. In the LIPIOJOINT-1 Trial, a 100% technical success rate was achieved with 73% of participants experiencing high improvement in pain, physical function, or both, at 3-month follow up [111]. Furthermore, the GRAVITY Trial is currently recruiting participants to evaluate the clinical, radiological, and biochemical effectiveness of GAE compared to conservative management with physical therapy [26].

### 11.2. Nerve Blocks and Ablations

In the wake of growing interest in minimally invasive procedures for knee OA treatment, nerve blocks and neurolytic therapies, such as radiofrequency ablation (RFA), have been proposed as additional alternatives to surgical interventions, demonstrating promising clinical outcomes [112,113,114,115,116,117,118,119,120,121,122]. A meta-analysis and literature review conducted by Sajan et al. in 2022 analyzed data from seven studies investigating GAE and thirteen studies examining RFA of the genicular nerve, medial retinacular nerve, infrapatellar branch of the saphenous nerve, and intra-articular nerves. The study compared patient-reported pain measurements using VAS at baseline and at various post-procedural intervals [123]. The analysis found that both RFA and GAE result in significant pain reduction post-procedurally [118]. Additionally, RFA demonstrated greater pain reduction at one-year follow-up, while GAE showed a higher reduction in pain during follow-ups within the first year after embolization [123]. Furthermore, GAE has been suggested to be a more cost-effective therapy compared to RFA [124]. Interestingly, a recent case report described the successful use of combined GAE and genicular nerve block as a treatment of chronic pain following total knee arthroplasty (TKA). The patient experienced improved functional status and pain relief over a 10-month follow-up period [125].

Currently, the Genicular Artery Embolization vs. Nerve Ablation Intervention (GENI) Trial is underway to evaluate the effectiveness of both GAE and phenol nerve ablation in OA patients, compared to a sham control group [21]. Additionally, another ongoing trial is assessing the utility of genicular nerve ablation prior to GAE, compared to GAE alone, in patients with chronic knee pain [20].

### 11.3. Intra-Articular Injections

The use of intra-articular injections for symptomatic relief of knee OA is a well-established therapy compared to GAE. Direct injection of various agents, such as corticosteroids, hyaluronic acid, and platelet-rich plasma, into the articular space at regular intervals has been shown to provide short-term symptomatic relief before considering surgical intervention [126,127]. However, various injection agents, and mixtures of agents, have been shown to provide variable efficacy and duration of effects when used for treating knee OA [128,129]. Presently, there are no data providing comparisons between specific injection agents (or specific combinations of agents) and GAE, leaving room for potential future directions. Nonetheless, a meta-analysis by Sajan et al. included intra-articular injections in their comparative analysis [123]. While intra-articular injections were found to be effective in improving symptoms, they were associated with the lowest reduction in reported pain and lacked the duration of symptomatic relief observed with RFA and GAE [123].

Further studies present conflicting evidence regarding the long-term therapeutic effects of intra-articular injections, but there are limited data suggesting they provide symptom relief as long-lasting as GAE [32,130,131,132,133,134]. Some data suggest that regimens of repeated injections at various scheduled intervals can provide improved pain that is sustained for one year after the last cycle of injections [135]. One study demonstrated that 73% of patients who underwent GAE discontinued intra-articular hyaluronic acid injections afterward [110].

Currently, the GAE Using Embosphere Microspheres vs. Corticosteroid Injections for Treatment of Symptomatic Knee OA (MOTION) trial is underway to compare the clinical outcomes and effectiveness of GAE versus intra-articular corticosteroid injections [24].

### 11.4. Partial and Total Knee Arthroplasty

As previously discussed, the current literature suggests that GAE may be an effective alternative for patients who are unsuitable surgical candidates and experience symptoms resistant to conservative management, effectively bridging the gap of invasivity between treatment options [44]. Some studies support the use of GAE in conjunction with surgical interventions, demonstrating beneficial clinical outcomes, such as reducing synovial hyperemia preoperatively in patients with hemophilia [136].

Additionally, GAE was initially introduced for the treatment of recurrent postoperative hemarthrosis following TKA and has shown clinical benefits compared to traditional invasive treatments, such as open or arthroscopic synovectomy [137,138,139]. The EPROGE Trial is currently recruiting participants to evaluate the benefits of using GAE as an adjunct therapy to improve pain and functionality after TKA [25]. Some studies also suggest that embolization before synovectomy may be beneficial, which can be extrapolated to the idea of preoperative genicular embolization in knee arthroplasty cases. This approach may serve as a prophylactic measure to prevent hemarthrosis in higher-risk surgical patients and further enhance postoperative pain relief and functional outcomes [57].

### 11.5. Economic and Cost-Effectiveness Analysis

The increasing prevalence of knee OA and the chronicity of the symptomatology results in a substantial economic burden globally, reaching costs in excess of USD 27 billion in the US [3]. In the lifetime of an individual diagnosed with knee OA, it has been estimated in a model that the medical costs directly attributed to this condition is USD 12,400, which increases substantially when eligibility for TKA is expanded [6,140]. Furthermore, conservative management with physical therapy and analgesia perform poorly in cost-effectiveness analyses, highlighting a demand for financially feasible bridging therapies [6,141,142].

Some studies have produced favorable cost-effectiveness analyses of minimally invasive procedures, such as RFA and intra-articular injections. One study demonstrated reduced overall healthcare costs and reduced costs associated with prescription analgesia in a cohort of patients that received intra-articular injection of hyaluronic acid, compared to cohorts of patients that received intra-articular injection of corticosteroids or TKA [143]. Another group of investigators has documented that RFA is highly cost-effective compared to intra-articular steroid injections and cost-effective compared to intra-articular hyaluronic acid injections over 6 months [144,145].

As mentioned, there is limited large-cohort research and high-quality evidence surrounding GAE for knee OA treatment, making reproducible and reliable cost-effectiveness comparisons challenging. The estimated baseline cost of a GAE procedure for knee OA is USD 3095.75, which is substantially more than conservative therapies and prescription analgesics, although nearly half of the estimated baseline cost of intra-articular hyaline injections [146,147]. A recently published cost-effectiveness study by Kwak et al. compared GAE and RFA to intra-articular corticosteroid injections [124]. In this study, GAE consistently demonstrated a higher cost-effectiveness probability compared to the other treatment modalities in a model simulating different cost-setting scenarios [124].

Once a larger cohort of data becomes available, it would be beneficial to perform similar comparative analyses comparing GAE to both conservative management as well as TKA, and to develop models comparing the cost-saving propensity of these therapies by delaying the need for surgical intervention.

## 12. Future Directions

Several potential future directions exist to further establish and develop the role of GAE in OA treatment. The development of newer embolic agents and improved embolization techniques could lead to better outcomes and potentially expand the eligible patient population, including those who are currently not considered suitable for GAE.

From a clinical perspective, stronger multidisciplinary collaborations between primary care, orthopedics, and interventional radiology could optimize OA management and position GAE as an earlier treatment option for patients with chronic knee OA pain who do not qualify for surgical intervention, regardless of OA severity (KL grades 1–4) [7,11,17]. Additionally, GAE could serve as an initial treatment for high-risk patients, such as those with a high BMI, to slow OA progression and delay the need for surgery [7].

Patients with severe, progressive OA who undergo GAE and later require surgical intervention should continue to be studied. However, preliminary evidence suggests that GAE does not increase the complication rates or the technical difficulty of surgery. Nonetheless, this concept should be evaluated further in longer-term, large-scale studies [17]. Currently, the longest post-procedure follow-up in the literature is two years [7,17,32]. Long-term follow-up studies would be highly beneficial in confirming the safety and reliability of GAE as an OA treatment and in determining the optimal timing and benefits of repeat procedures.

GAE may also be a viable alternative treatment for various other knee conditions and injuries. Initial evidence suggests promising results in the treatment of overuse sports injuries, such as pes anserine tendinopathy and patellar tendinopathy. As a minimally invasive procedure with a shorter recovery time compared to conventional conservative or surgical treatments, GAE is an attractive therapeutic option. Currently, available evidence is limited to case studies in elite athletes, but further research could lay the groundwork to expand the indications to sports-related injuries [148].

New imaging modalities, such as optical coherence tomography (OCT), have been explored and may improve initial patient assessment, patient selection, and response evaluation during follow-up [149,150,151].

Finally, genomic, transcriptomic, proteomic, and metabolomic insights into OA pathophysiology could further optimize patient selection. A better understanding of the abnormal signaling pathways and genetic/epigenetic regulatory factors underlying cartilage destruction, pain, and disease progression may enable personalized treatment strategies and an improved prediction of individual patient response to GAE [63,152].

## 13. Conclusions

Genicular artery embolization (GAE) has emerged as a promising minimally invasive treatment for osteoarthritis (OA), offering pain relief and improved function for patients who are not ideal candidates for surgery or as a bridge to delay surgical intervention. As the prevalence of OA and obesity continues to rise, the need for alternative treatment options beyond total knee arthroplasty (TKA) is becoming increasingly critical. Early research has demonstrated that GAE can reduce pain and improve mobility, with sustained benefits lasting up to two years after embolization.

The evolution of GAE from its initial use in treating postoperative hemarthrosis to its current role in OA management highlights the growing recognition of vascular contributions to OA pathophysiology. By selectively embolizing hypervascular genicular arteries, GAE disrupts the inflammatory cascade that drives pain and joint degeneration, setting it apart from traditional therapies such as intra-articular injections and nerve ablations. Multiple ongoing randomized controlled trials are currently evaluating GAE’s safety and efficacy, with more robust outcome measures and higher-quality evidence expected in the near future.

Despite its promising results, several challenges remain. The long-term durability of GAE beyond two years is not yet well established, and further studies are needed to optimize patient selection criteria.

Future advancements in embolic materials, imaging techniques, and molecular profiling may help refine patient selection, further assess GAE’s effectiveness, and expand its indications beyond OA to other musculoskeletal conditions, such as sports-related injuries. With ongoing research and multidisciplinary collaboration, GAE has the potential to become a standard therapeutic option in the continuum of OA care, providing relief for patients seeking alternatives to surgery while maintaining or improving their quality of life.

## Figures and Tables

**Table 1:** A summary of registered clinical trials investigating GAE for OA ^1^.

Timeline	Trial Name (Brief Title)	Principal Investigator, Affiliation	Status	Sponsor	Location	Trial Type	Inclusion Criteria	Enrollment (Actual or Anticipated)
Jun 2012–Dec 2013	Transcatheter Arterial Embolization as a Treatment for Medial Knee Pain in Patients with Mild to Moderate Osteoarthritis	Yuji Okuno, Edogawa Hospital [10]	Completed	None	Tokyo, Japan	Single-center, single-arm, prospective pilot study.	- Moderate-to-severe medial knee pain VAS > 50 mm resistant to at least 3 months of conservative therapies - KL grade 1–2	14
Jun 2012–Sep 2014	Midterm Clinical Outcomes and MR Imaging Changes after Transcatheter Arterial Embolization as a Treatment for Mild to Moderate Radiographic Knee Osteoarthritis Resistant to Conservative Treatment	Yuji Okuno, Edogawa Hospital [11]	Completed	None	Tokyo, Japan	Prospective, single-center, single-arm pilot study.	- Presence of knee pain - KL grade 1–3 assessed by routine weight-bearing knee radiographs - Local tenderness around the knee - Patient age 40–80 years - 3 months or more of conservative therapies and persistent moderate-to-severe knee pain (VAS score > 50 mm)	72
May 2018–Apr 2020	Geniculate Artery Embolization for Knee Pain Secondary to Osteoarthritis (OA)	Ari Isaacson MD, University of North Carolina, Chapel Hill [12]	Completed	Medtronic & University of North Carolina, Chapel Hill	Chapel Hill, NC, USA, and Woodbridge, VA, USA	Single-blinded, multicenter, randomized-controlled study of GAE versus placebo sham procedure.	- Moderate-to-severe knee pain (visual analog scale (VAS) > 50 mm) - Pain refractory to at least 3 months of conservative therapies - KL grade 1–3	21
Feb 2019–Oct 2021	Genicular Artery Embolization for the Treatment of Knee Osteoarthritis (GAE-OA)	Siddharth Padia MD, University of California, Los Angeles [15]	Completed	Boston Scientific Corporation	Santa Monica, CA, USA	Prospective, single-center, single-arm, phase 2 investigational study.	- Age ≥ 40 years and ≤80 years - Life expectancy greater than 12 months - Ineligibility for or refusal of surgical management - Moderate-to-severe knee pain as determined by visual analog scale > 4 - Osteoarthritis based on X-ray - Local knee tenderness - Resistant/failed conservative treatment	40
Feb 2020–May 2024	Genicular Artery embolisation in Patients with Osteoarthritis of the Knee (GENESIS)	Mark W. Little MD, Royal Berkshire NHS Foundation Trust [17]	Completed	Merit Medical Systems Inc (USA)	Reading, UK	Single-center, single-arm prospective pilot study.	- Patients 45 years or older with mild-to-moderate knee osteoarthritis defined as Kellgren–Lawrence (KL) grade 1–3 on plain X-ray -Knee pain for over 6 months despite conservative management	38
Sep 2022–Dec 2023	Genicular Artery Embolization as Pain Treatment of Knee Osteoarthritis (GETKO)	Professor Lars Lönn, University Hospital of Copenhagen, Denmark [33]	Completed	Rigshospitalet, Denmark	Copenhagen, Denmark	Single-center, single-arm, prospective pilot study.	- Body mass index < 35 kg/m^2^ - X-ray verified mild-to-moderate knee OA (KL grade 1–3), obtained maximum 6 months prior to inclusion - Moderate-to-severe knee pain during walking (VAS > 50 mm) resistant to minimum 3 months physiotherapy	17
Dec 2022–Dec 2025	Effectiveness and Safety of Embosphere Microspheres for Embolization of the Geniculate Artery for the Treatment of Pain With Known Moderate to Severe Knee Osteoarthritis	Shivank Bhatia, University of Miami [34]	Recruiting	None.	Miami, FL, USA	Single-center, single-arm, prospective interventional study to assess effectiveness and safety of embosphere microspheres in GAE for OA.	- Subject is age 40–80 - Subject is able to have an MRI - Minimum of prior 12 weeks of failed response to conservative therapy for knee osteoarthritis - Localized tenderness in anterior knee area - KL grade 1–3 - Synovitis present as assessed by WORMS - VAS > 50 mm - WOMAC score > 30	16
Jan 2023–Jun 2024	Geniculate Artery Embolization for the Treatment of Knee Osteoarthritis	Jason Wong MD, University of Calgary [35]	Recruiting	University of Calgary	Calgary, AB, Canada	Single-center, single-arm, prospective interventional study.	- VAS score of at least 50 mm for knee pain - Pain resistant to at least 3 months of conservative therapy - Age > 40 years - Radiographs demonstrating knee osteoarthritis on same side as pain - Patient not a surgical candidate or declines surgical management	50
Dec 2023–Aug 2024	First in Human Study of SakuraBead for Genicular Artery Embolization to Treat Pain Secondary to Knee Osteoarthritis	Not listed [30]	Completed	Crannmed	Tashkent, Uzbekistan	Prospective, single-arm, unmasked, first in human study of the safety and efficacy of SakuraBead microspheres in GAE for OA.	- Clinical diagnosis of knee OA - Moderate-to-severe knee pain (VAS ≥ 5) - Pain refractory to at least 3 months of conservative therapies - KL grade 1–3 on radiograph of the knee - Age 40 years or older - Confirmed evidence of knee OA, defined as an angiographic ‘blush’ pattern in one or more of the target genicular artery(ies)	15
Dec 2021–Jun 2026	Geniculate Artery Embolization for Treatment of Osteoarthritis	Bedros Taslakian MD, NYU Langone Health [28]	Active, not recruiting	NYU Langone Health	New York, NY, USA	Single-center, single-arm, prospective interventional pilot study.	- 30–80 years of age of any gender - KL Grade 2–4 knee OA on most recent knee radiograph obtained within 6 months of screening visit - Knee pain resistant to conservative treatment for at least 3 months - Moderate-to-severe knee pain VAS ≥ 40 mm	38
Oct 2022–Jun 2028	Genicular Artery Embolisation for Knee Osteoarthritis II (GENESIS II)	Mark W. Little MD, Royal Berkshire NHS Foundation Trust [23]	Recruiting	Varian, A Siemens Healthineers Company	Reading, UK	Single-center, randomized control trial comparing GAE versus sham procedure.	- Participants aged 45 years or above - Grade 1–3 knee OA on X-ray as per KL Grading Scale - Knee pain for at least 3 months resistant to conservative non-surgical treatment - Be able to lie flat for at least 6 h; this will be assessed by asking how participants sleep and assessing what prevents them from lying flat overnight - Minimum score of 50 on baseline 0–100 VAS	110
Dec 2023–Dec 2026	Genicular Artery Embolization vs. Nerve Ablation Intervention (GENI) for Knee Osteoarthritis	David Clinkard MD, Alexandre Menard MD, Steve Mann MD, Queen’s University [21]	Recruiting	Queen’s University	Kingston, ON, Canada	Single-center, randomized control trial comparing GAE, genicular nerve phenol ablation, and sham procedure.	- Age > 40 years - Knee pain due to osteoarthritis for at least 6 months - Pain refractory to conservative therapies for at least 3 months with a desire for TKA	150
Aug 2023–Oct 2027	Genicular Artery Embolization Vs Observation for Symptomatic Knee Osteoarthritis (GRAVITY)	Siddharth Padia MD, University of California, Los Angeles [26]	Recruiting	Varian Medical Systems	Santa Monica, CA, USA	Single-center, prospective, open-label, randomized control trial comparing GAE versus observation.	- Age ≥ 40 years and less than 80 years - Ineligibility for or refusal of surgical management - Moderate-to-severe knee pain as determined by VAS > 5 out of 10 - Osteoarthritis based on X-ray; KL score > 2 based on radiograph completed within 3 months of procedure date - Resistant/failed conservative treatment for at least 3 months	100
Oct 2023–Oct 2028	Creation of a Prospective Data Collecting Registry for Genicular Artery Embolization for Arthritis (GAE)	Osmannudin Ahmed MD, University of Chicago [36]	Recruiting	University of Chicago	Chicago, IL, USA	Prospective observational patient registry.	- Aged ≥ 18 - Bilateral or unilateral knee pain attributed to knee osteoarthritis (KOA). For bilateral KOA patients, the more severe knee will be permitted inclusion to the registry - Grade 1–3 Osteoarthritis as diagnosed on KL Grading scale - Knee pain > 6 months refractory to conservative medical management - Not eligible for surgical knee replacement or patient’s personal preference to undergo GAE	100
Sep 2024–Apr 2025	Sequential Genicular Nerve Ablation Prior to Geniculate Artery Embolization in Knee Pain	Ahmed Bessar MD PhD, Zagazig University [20]	Recruiting	Zagazig University	Zagazig, Egypt	Single-center, randomized control trial evaluating effectiveness of sequential genicular nerve ablation followed by GAE versus GAE alone.	- Adults aged 18 to 80 years - Diagnosed with chronic knee pain refractory to conservative treatment	60
Nov 2024–Apr 2027	SakuraBead Used As Resorbable Embolic for Genicular Artery Embolization (SURE)	Not listed [29]	Recruiting	CrannMed	Raleigh, NC, USA, and Tashkent, Uzbekistan	Open label, prospective, two-arm, multicenter randomized control trial comparing SakuraBead GAE with control steroid injections.	- Clinical diagnosis of knee OA - Moderate-to-severe knee pain (WOMAC Pain ≥ 10) - Pain refractory to at least 3 months of conservative therapy - KL grade 1–3 - Age ≥ 40 years and <80 years	89
Jan 2024–Oct 2027	GAE Using Embosphere Microspheres Vs Corticosteroid Injections for Treatment of Symptomatic Knee OA (MOTION)	Sandeep Bagla MD, Prostate Centers, USA Craig J McAsey MD, Anderson Orthopedic Clinic [24]	Recruiting	Merit Medical Systems, Inc.	22 locations across the USA, Australia, Brazil, Canada, New Zealand, and the UK	Multicenter, prospective, interventional trial comparing outcomes of GAE versus steroid injections.	- Age ≥ 21 years - Mild-to-severe knee pain, defined as a WOMAC Pain score of ≥8 out of 20 (in the target knee) - Pain refractory to conservative therapies for at least 90 days prior to enrollment/randomization - KL grade 1–4	264
Sept 2024–Oct 2028	Effect of Genicular Arteries Embolization in Symptomatic Knee Osteoarthritis LipioJoint-2 (LIPIOJOINT-2)	Marc Sapoval MD, Hôpitaux de Paris [22]	Recruiting	Assistance Publique–Hôpitaux de Paris	Paris, France	Single-blind, multicenter, phase 3 randomized control trial comparing GAE using ethiodized oil-based emulsion versus sham control procedure.	- Diagnosis of primary KOA according to the classification of the American College of Rheumatology (ACR) - Radiographic KL score ≥ 2 - -VAS pain score ≥ 40 mm - Previous intra-articular injection in the target knee - Patient not eligible to knee surgery - For women of childbearing potential: negative bêta-HCG before randomization	130
Jul 2024–Jul 2029	Embo Registry; National Registry for Artery Embolization	Siddhartha Rao MD, Vascular Solutions of North Carolina [37]	Recruiting	Vascular Solutions of North Carolina	Cary, NC, USA, and Winchester, TN, USA	Retrospective and prospective observational patient registry.	- Patients >18 years of age having undergone artery embolization interventions for the treatment of chronic pain due to osteoarthritis or other diagnoses that cause localized pain	1000
Jan 2025–Nov 2026	Genicular Artery Embolization (GAE) for Osteoarthritic Knee Pain	Andrew C. Picel MD, Stanford University [38]	Recruiting	N/A	Palo Alto, CA, USA	Single-arm, prospective pilot study.	- Age ≥ 40 years - Moderate-to-severe knee pain (VAS > 40 mm) - Pain refractory to 3 months of conservative treatments - KL radiographic grade 1–3 disease - MRI features of active synovitis - Ineligibility or refusal of surgical management - Local knee tenderness	30
Jan 2025–Feb 2027	Efficacy of Microparticle Geniculate Artery Embolization in Total Knee Prosthesis Patients with Pain Resistant to Medical Treatment. a Prospective Randomized Controlled Trial (EPROGE)	Christian Roux MD, Centre Hospitalier Universitaire de Nice [25]	Not Yet Recruiting	Societe Francaise de Rhumatologie	11 locations across France	Double-blind, multicenter, randomized control trial comparing GAE versus sham procedure.	- Aged between 40 and 80 years with a TKR for more than one year - TKR pain with a VAS ≥ 40 mm having progressed for at least 3 months despite the initiation of a well-conducted medical treatment - Investigations within 6 months to rule out malposition, loosening, and sepsis - No revision surgery envisaged - Echodoppler hyperemia over the painful area of the knee	112

^1^ Source: www.clinicaltrials.gov (accessed on 14 February 2025).

**Table 2:** Suggested contraindications to GAE with rationales, potential associated complications, and risk minimization strategies.

Contraindication	Rationale and Potential Complication(s)	Strategies to Minimize Risk of Complication
Severe Peripheral Arterial Disease	- The genicular arteries provide collateral perfusion of the distal lower extremity in patients with PAD. - Occlusion of the genicular arteries during embolization may cause worsening of the vascular disease, leading to pain, non-healing wounds, and possibly even critical limb ischemia.	- Ensure patient optimization with adequate treatment of peripheral arterial disease pre-procedurally. - Achieve as distal a cannulation into target vessels as possible to minimize the risk of off-target embolization.
Active or Suspected Knee Infection	- Embolization of the genicular arteries during an active infection prohibits the physiologic healing process and may exacerbate the infection, with the potential of progression to more severe infection such as osteomyelitis.	- Perform thorough physical exam before procedure to evaluate for infectious signs/symptoms. - Use routine pre-procedural antibiotics (e.g., 2 g IV cefazolin). - Consider adequate course of antibiotics if suspicion for infection arises.
Renal Dysfunction	- Patients with renal dysfunction may be at increased risk of contrast induced nephrotoxicity.	- Encourage oral and/or intravenous hydration before and after procedure to promote extracellular volume expansion. - Withhold nephrotoxic medications as able. - Use the lowest amount of contrast dye as possible intraoperatively. - Consider CO_2_ angiography if able.
Normal Radiographic Knee Joint	- An alternative diagnosis should be considered, as GAE may not provide any therapeutic benefit in alternative pathologies.	- Ensure comprehensive review of patient medical history, biochemical markers, and prior imaging.
Fibromyalgia, Autoimmune or Inflammatory Disorder	- An alternative diagnosis should be considered, as GAE _may_ not provide any therapeutic benefit in alternative pathologies.	- Ensure comprehensive review of patient medical history, biochemical markers, and prior imaging.

**Table 3:** Pre-treatment evaluation tools prior to genicular artery embolization (GAE) for knee osteoarthritis (OA).

Clinical Information	Reasoning
Pain and quality of life	Baseline pain score and quality of life to assess suitability for procedure and allow for evaluation of clinical outcomes postoperatively.
Body mass index (BMI)	Risk assessment and pre-procedural planning.
PAD ^1^/risk factors for PAD	Risk assessment and pre-procedure planning.
Knee radiograph	Baseline level of degeneration and as an initial comparison for radiographic follow-up after procedure. Also allows for some anatomical considerations in pre-procedure planning.
Knee MRI ^2^	Pre-procedure technical planning, allowing for assessment of relevant vascular anatomy, potential embolization targets, and the presence of anatomical variations.

^1^ Peripheral arterial disease. ^2^ Magnetic resonance imaging.

**Table 5:** Proposed genicular anastomosis anatomical variant classification systems and frequency of observation [82,83].

Sighary et al. Classification System			Callese et al. Classification System		
Sighary Classification	Description	Frequency	Callese Classification	Description	Frequency
**Descending Genicular Artery (DGA)**					
**Type A**	Bifurcation of the saphenous and osteoarticular/muscular branches occurs **above** the midpoint between the origin of the DGA and the superior aspect of the medial femoral condyle.	24%	**Type A**	Two branches of the DGA with deep (osteoarticular) and superficial (myocutaneous) termini.	77%
**Type B**	Bifurcation of the saphenous and osteoarticular/muscular branches occurs **below** the midpoint between the origin of the DGA and the superior aspect of the medial femoral condyle.	72%	**Type B**	Single vessel with no branching.	17%
**Type C**	**Separate** origins of the saphenous and osteoarticular/muscular branches.	4%	**Type C**	Diminutive vessel.	5%
			**Type D**	Absent vessel.	1%
**Genicular Arteries**					
**Type I**	Independent branching of the SMGA, SLGA, MGA, IMGA, and ILGA.	28%	**Type 1**	MGA and SLGA share common origin, with independent branching of the remaining arteries (Sighary Type III).	56.1%
**Type II**	MGA and SMGA share common origin, with independent branching of the remaining arteries.	22%	**Type 2**	Independent branching of the SMGA, SLGA, MGA, IMGA, and ILGA (Sighary Type I).	21.9%
**Type III**	MGA and SLGA share common origin, with independent branching of the remaining arteries.		**Type 3**	SLGA, SMGA, and MGA share common origin, with independent branching of the inferior genicular arteries (Sighary Type V).	15.6%

**Table 6:** Recommendations for pre-procedural management of anticoagulation and antiplatelet medications in low- and high-bleeding-risk procedures [92,93,94,95,96,97].

Medication	Low Risk of Bleeding	High Risk of Bleeding
Aspirin	Do not hold	Withhold 3–5 days before procedure. Resume post-op day (POD)1.
Aspirin/Dipyridamole/Aggrenox	Do not hold	Withhold 3–5 days before procedure Resume POD1.
Short-acting NSAIDs (half-life 2–6 h): diclofenac, ketoprofen, indomethacin, ketorolac, ibuprofen	Do not hold	No recommendation.
Intermediate-acting NSAIDs (half-life 7–15 h): naproxen, sulindac, diflunisal, celecoxib	Do not hold	No recommendation.
Long-acting NSAIDs (half-life > 20 h): meloxicam, nabumetone, piroxicam	Do not hold	No recommendation.
Unfractionated heparin	Do not hold	Withhold IV heparin for 4–6 h before procedure; check aPTT or anti-Xa level; for BID or TID dosing of SC heparin, procedure may be performed 6 h after the last dose. Resume: 6–8 h.
Low-molecular-weight heparin (LMWH): enoxaparin (Lovenox), dalteparin (Fragmin)	Do not hold	Enoxaparin: withhold 1 dose if prophylactic dose is used; withhold 2 doses or 24 h before procedure if therapeutic dose is used; check anti-Xa level if renal function impaired; For dalteparin, withhold 1 dose before procedure. Resume: 12 h.
Fondaparinux (Arixtra)	Do not hold	Withhold 2/3 d (CrCl ≥ 50 mL/min) or 3–5 d (CrCl ≤ 50 mL/min). Resume: 24 h.
Argatroban (Acova)	Do not hold	Withhold 2–4 h before the procedure; check aPTT. Resume: 4–6 h.
Bivalirudin (Angiomax)	Do not hold	Withhold 2–4 h before the procedure; check aPTT. Resume: 4–6 h.
Warfarin (Coumadin)	Target INR 3.0; consider bridging for high-thrombosis-risk cases Restart: same day for bridging patients	Withhold 5 d until target INR 1.8; consider bridging for high thrombosis risk cases. If STAT or emergent, use a reversal agent. Resume: POD1, or multidisciplinary, shared decision making recommended if vitamin K, reversal agent, or bridging with LMWH.
Apixaban (Eliquis)	Do not hold	Withhold 4 doses (CrCl ≥ 50 mL/min) or 6 doses (CrCl < 30–50 mL/min). If the procedure is STAT, use a reversal agent (andexanet alfa); consider checking anti-Xa activity or apixaban level if impaired renal function. Resume: 24 h.
Betrixaban (Bevyxxa)	Do not hold	Withhold for 3 doses. If the procedure is STAT, use a reversal agent (andexanet alfa); consider checking anti-Xa activity with impaired renal function. Resume: 24 h.
Dabigatran (Pradaxa)	Do not hold	Withhold 4 doses (CrCl >50 mL/min) or 6–8 doses (CrCl < 30–50 mL/min); if procedure is STAT, use reversal agent (idarucizumab); consider checking thrombin time or dabigatran level with impaired renal function. Resume: 24 h.
Edoxaban (Savaysa)	Do not hold	Withhold for 2 doses; if procedure is STAT or emergent, use reversal agent (andexanet alfa); consider checking anti-Xa activity with impaired renal function. Resume: 24 h.
Rivaroxaban (Xarelto)	Do not hold	Defer procedure until off medication for 2 doses (CrCl > 50 mL/min), 2 doses (CrCl < 30–50 mL/min), or 3 doses (CrCl < 15–30 mL/min); if procedure is STAT, use reversal agent (andexanet alfa); consider checking anti-Xa activity or rivaroxaban level with impaired renal function. Resume: 24 h.
Clopidogrel (Plavix)	Do not hold	Withhold for 5 d before the procedure. Resume: 6 h after procedure if using 75 mg or 24 h after procedure if using a loading dose (300–600 mg).
Ticagrelor (Brilinta)	Do not hold	Withhold for 5 d before the procedure. Resume: POD1.
Prasugrel (Effient)	Do not hold	Withhold for 7 d before the procedure. Resume: POD1.
Cangrelor (Kengreal)	Defer procedure until off medication; if procedure is STAT, withhold 1 h before procedure; Resume: multidisciplinary discussion with cardiology suggested.	Multidisciplinary, shared decision making recommended.
Cilostazol (Pletal)	Do not hold	Do not hold
Short-acting: eptifibatide (Integrilin), tirofiban (Aggrastat)	Hold 4–8 h before procedure	Multidisciplinary, shared decision making recommended.
Antiplatelet agents: glycoprotein IIb/IIIa inhibitors Long-acting abciximab (ReoPro)	Hold 24 h before procedure	Multidisciplinary, shared decision making recommended.

## Abbreviations

The following abbreviations are used in this manuscript:

GAE	Genicular artery embolization
OA	Osteoarthritis
TKA	Total knee arthroplasty
KOOS	Knee injury and osteoarthritis outcome score
WOMAC	Western Ontario and McMaster universities arthritis index
VAS	Visual analog scale
RFA	Radiofrequency ablation
BMI	Body mass index
PAD	Peripheral arterial disease
KL	Kellgren–Lawrence scale
WORMS	Whole-organ magnetic resonance imaging score
OAKHQOL	Osteoarthritis knee and hip quality of life
OA-QoL	Osteoarthritis quality of life
VEGF	Vascular Endothelial Growth Factor
Ang-1	Angiopoietin-1
DNA	Deoxyribonucleic acid
miRNA	Micro-ribonucleic acid
NGF	Nerve growth factor
CGRP	Calcitonin gene-related peptide
CL2/CCR2	Chemokine C-C motif ligand 2 and receptor 2
TNFα	Tumor necrosis factor alpha
PA	Popliteal artery
DGA	Descending genicular artery
SB	Saphenous branch
MB	Muscular branch
OAB	Osteoarticular branch
SMGA	Superior medial genicular artery
MGA	Middle genicular artery
SLGA	Superior lateral genicular artery
IMGA	Inferior medial genicular artery
ILGA	Inferior lateral genicular artery
ATRA	Anterior tibial recurrent artery
ATA	Anterior tibial artery
OCT	Optical coherence tomography
CBCT	Cone-beam computerized tomography
FDA	Food and Drug Administration
IPM-CS	Imipenem/cilastatin
PVA	Polyvinyl alcohol
DSA	Digital subtraction angiography
POD	Postoperative day
CrCl	Creatinine clearance
NSAID	Non-steroidal anti-inflammatory drug
DOAC	Direct oral anticoagulant
ASA	American Society of Anesthesiologists

## Appendix A

**Table A1:** Knee Injury and Osteoarthritis Outcomes Score Questionnaire Knee Injury and Osteoarthritis Outcome Score (KOOS).

Pain					
P1 How often is your knee painful?	▢ Never	▢ Monthly	▢ Weekly	▢ Daily	▢ Always
What degree of pain have you experienced the last week when…?					
P2 Twisting/pivoting on your knee	▢ None	▢ Mild	▢ Moderate	▢ Severe	▢ Extreme
P3 Straightening knee fully	▢ None	▢ Mild	▢ Moderate	▢ Severe	▢ Extreme
P4 Bending knee fully	▢ None	▢ Mild	▢ Moderate	▢ Severe	▢ Extreme
P5 Walking on flat surface	▢ None	▢ Mild	▢ Moderate	▢ Severe	▢ Extreme
P6 Going up or down stairs	▢ None	▢ Mild	▢ Moderate	▢ Severe	▢ Extreme
P7 At night while in bed	▢ None	▢ Mild	▢ Moderate	▢ Severe	▢ Extreme
P8 Sitting or lying	▢ None	▢ Mild	▢ Moderate	▢ Severe	▢ Extreme
P9 Standing upright	▢ None	▢ Mild	▢ Moderate	▢ Severe	▢ Extreme
Symptoms					
Sy1 How severe is your knee stiffness after first wakening in the morning?	▢ None	▢ Mild	▢ Moderate	▢ Severe	▢ Extreme
Sy2 How severe is your knee stiffness after sitting, lying, or resting later in the day?	▢ None	▢ Mild	▢ Moderate	▢ Severe	▢ Extreme
Sy3 Do you have swelling in your knee?	▢ Never	▢ Rarely	▢ Sometimes	▢ Often	▢ Always
Sy4 Do you feel grinding, hear clicking or any other type of noise when your knee moves?	▢ Never	▢ Rarely	▢ Sometimes	▢ Often	▢ Always
Sy5 Does your knee catch or hang up when moving?	▢ Never	▢ Rarely	▢ Sometimes	▢ Often	▢ Always
Sy6 Can you straighten your knee fully?	▢ Always	▢ Often	▢ Sometimes	▢ Rarely	▢ Never
Sy7 Can you bend your knee fully?	▢ Always	▢ Often	▢ Sometimes	▢ Rarely	▢ Never
Activities of daily living					
What difficulty have you experienced the last week…?					
A1 Descending	▢ None	▢ Mild	▢ Moderate	▢ Severe	▢ Extreme
A2 Ascending stairs	▢ None	▢ Mild	▢ Moderate	▢ Severe	▢ Extreme
A3 Rising from sitting	▢ None	▢ Mild	▢ Moderate	▢ Severe	▢ Extreme
A4 Standing	▢ None	▢ Mild	▢ Moderate	▢ Severe	▢ Extreme
A5 Bending to floor/picking up an object	▢ None	▢ Mild	▢ Moderate	▢ Severe	▢ Extreme
A6 Walking on flat surface	▢ None	▢ Mild	▢ Moderate	▢ Severe	▢ Extreme
A7 Getting in/out of car	▢ None	▢ Mild	▢ Moderate	▢ Severe	▢ Extreme
A8 Going shopping	▢ None	▢ Mild	▢ Moderate	▢ Severe	▢ Extreme
A9 Putting on socks/stockings	▢ None	▢ Mild	▢ Moderate	▢ Severe	▢ Extreme
A10 Rising from bed	▢ None	▢ Mild	▢ Moderate	▢ Severe	▢ Extreme
A11 Taking off socks/stockings	▢ None	▢ Mild	▢ Moderate	▢ Severe	▢ Extreme
A12 Lying in bed (turning over, maintaining knee position)	▢ None	▢ Mild	▢ Moderate	▢ Severe	▢ Extreme
A13 Getting in/out of bath	▢ None	▢ Mild	▢ Moderate	▢ Severe	▢ Extreme
A14 Sitting	▢ None	▢ Mild	▢ Moderate	▢ Severe	▢ Extreme
A15 Getting on/off toilet	▢ None	▢ Mild	▢ Moderate	▢ Severe	▢ Extreme
A16 Heavy domestic duties (shovelling, scrubbing floors, etc.)	▢ None	▢ Mild	▢ Moderate	▢ Severe	▢ Extreme
A17 Light domestic duties (cooking, dusting, etc.)	▢ None	▢ Mild	▢ Moderate	▢ Severe	▢ Extreme
Sport and recreation function					
What difficulty have you experienced the last week…?					
Sp1 Squatting	▢ None	▢ Mild	▢ Moderate	▢ Severe	▢ Extreme
Sp2 Running	▢ None	▢ Mild	▢ Moderate	▢ Severe	▢ Extreme
Sp3 Jumping	▢ None	▢ Mild	▢ Moderate	▢ Severe	▢ Extreme
Sp4 Turning/twisting on your injured knee	▢ None	▢ Mild	▢ Moderate	▢ Severe	▢ Extreme
Sp5 Kneeling	▢ None	▢ Mild	▢ Moderate	▢ Severe	▢ Extreme
Knee-related quality of life					
Q1 How often are you aware of your knee problems?	▢ Never	▢ Monthly	▢ Weekly	▢ Daily	▢ Always
Q2 Have you modified your lifestyle to avoid potentially damaging activities to your knee?	▢ Not at all	▢ Mildly	▢ Moderately	▢ Severely	▢ Totally
Q3 How troubled are you with lack of confidence in your knee?	▢ Not at all	▢ Mildly	▢ Moderately	▢ Severely	▢ Totally
Q4 In general, how much difficulty do you have with your knee?	▢ None	▢ Mild	▢ Moderate	▢ Severe	▢ Extreme

**Table A2:** The Western Ontario and McMaster Universities Osteoarthritis Index Questionnaire.

The Western Ontario and McMaster Universities Osteoarthritis Index (WOMAC)						
Name:						
Date:						
Instructions: Please rate the activities in each category according to the following scale of difficulty:						
0 = None, 1 = Slight, 2 = Moderate, 3 = Very, 4 = Extremely						
Circle **one number** for each activity						
Pain	1. Walking	0	1	2	3	4
	2. Stair Climbing	0	1	2	3	4
	3. Nocturnal	0	1	2	3	4
	4. Rest	0	1	2	3	4
	5. Weight bearing	0	1	2	3	4
Stiffness	1. Morning stiffness	0	1	2	3	4
	2. Stiffness occurring later in the day	0	1	2	3	4
Physical Function	1. Descending stairs	0	1	2	3	4
	2. Ascending stairs	0	1	2	3	4
	3. Rising from sitting	0	1	2	3	4
	4. Standing	0	1	2	3	4
	5. Bending to floor	0	1	2	3	4
	6. Walking on flat surface	0	1	2	3	4
	7. Getting in/out of car	0	1	2	3	4
	8. Going shopping	0	1	2	3	4
	9. Putting on socks	0	1	2	3	4
	10. Lying in bed	0	1	2	3	4
	11. Taking off socks	0	1	2	3	4
	12. Rising from bed	0	1	2	3	4
	13. Getting in/out of bath	0	1	2	3	4
	14. Sitting	0	1	2	3	4
	15. Getting on/off toilet	0	1	2	3	4
	16. Heavy domestic duties	0	1	2	3	4
	17. Light domestic duties	0	1	2	3	4
Total Score: /96 = %						
Comments/Interpretation (to be completed by therapist only):						
